# An international, phase III randomized trial in patients with mucinous epithelial ovarian cancer (mEOC/GOG 0241) with long-term follow-up: and experience of conducting a clinical trial in a rare gynecological tumor

**DOI:** 10.1016/j.ygyno.2019.03.256

**Published:** 2019-06

**Authors:** Martin Gore, Allan Hackshaw, William E. Brady, Richard T. Penson, Richard Zaino, W. Glenn McCluggage, Raji Ganesan, Nafisa Wilkinson, Timothy Perren, Ana Montes, Jeffrey Summers, Rosemary Lord, Graham Dark, Gordon Rustin, Melanie Mackean, Nicholas Reed, Sean Kehoe, Michael Frumovitz, Helen Christensen, Amanda Feeney, Jonathan Ledermann, David M. Gershenson

**Affiliations:** a Royal Marsden NHS Foundation Trust, London, UK; b Cancer Research UK & UCL Cancer Trials Centre, London, UK; c Roswell Park Comprehensive Cancer Centre, Buffalo, USA; d Massachusetts General Hospital, Boston, USA; e Penn State Health Milton S. Hershey Medical Centre, PA, USA; f Dept of Pathology, Belfast Health & Social Care Trust, Belfast, UK; g Birmingham Women's Hospital, Birmingham, UK; h University College London Hospitals NHS Foundation Trust, London, UK; i Leeds Teaching Hospitals NHS Trust, Leeds, UK; j Guy's and St Thomas' NHS Foundation Trust, London, UK; k Maidstone and Tunbridge Wells NHS Foundation Trust, Kent, UK; l Clatterbridge Cancer Centre NHS Foundation Trust, Liverpool, UK; m Newcastle upon Tyne Hospitals NHS Foundation Trust, Newcastle upon Tyne, UK; n Mount Vernon Cancer Centre, Middlesex, UK; o Edinburgh Cancer Centre, Edinburgh, UK; p Beatson Oncology Centre, Glasgow, UK; q Institute of Cancer and Genomics, University of Birmingham, Birmingham, UK; r The University of Texas MD Anderson Cancer Centre, Houston, TX, USA

**Keywords:** Mucinous ovarian cancer, Chemotherapy, Rare tumor trial, Factorial design

## Abstract

**Objectives:**

We evaluated four different treatment regimens for advanced-stage mucinous epithelial ovarian cancer.

**Methods:**

We conducted a multicenter randomized factorial trial (UK and US). Patients were diagnosed with primary mEOC: FIGO stage II–IV or recurrence after stage I disease. Treatment arms were paclitaxel-carboplatin, oxaliplatin-capecitabine, paclitaxel-carboplatin-bevacizumab, or oxaliplatin-capecitabine-bevacizumab. Chemotherapy was given 3-weekly for 6 cycles, and bevacizumab (3-weekly) was continued as maintenance (for 12 cycles). Endpoints included overall-survival (OS), progression-free survival (PFS), toxicity and quality of life (QoL).

**Results:**

The trial stopped after 50 patients were recruited due to slow accrual. Median follow-up was 59 months. OS hazard ratios (HR) for the two main comparisons were: 0.78 (*p* = 0.48) for Oxal-Cape vs. Pac-Carbo (each with/without bevacizumab), and 1.04 (*p* = 0.92) for bevacizumab vs. no bevacizumab. Corresponding PFS HRs were: 0.84 and 0.80. Retrospective central pathology review revealed only 45% (18/40) cases with available material had confirmed primary mEOC. Among these, OS HR for Oxal-Cape vs. Pac-Carbo was 0.36 (*p* = 0.14); PFS HR = 0.62 (*p* = 0.40). Grade 3–4 toxicity was seen in 61% Pac-Carbo, 61% Oxal-Cape, 54% Pac-Carbo-Bev, and 85% Oxal-Cape-Bev. QoL was similar between the four arms.

**Conclusion:**

mEOC/GOG0241 represents an example of a randomized rare tumor trial. Logistical challenges led to early termination, including difficulties in local histopathological diagnosis and accessing drugs outside their labelled indication. There was misalignment between central funders who support clinical trials in rare cancers and the deprioritisation of such work by those managing and funding research at a local level. Rare cancer trials should include centralised pathology review before treatment.

Clinical trial registry number: ISRCTN83438782.

## Highlights

• mEOC/GOG-0241 is one of the first international rare tumor trials.
• Oxaliplatin/capecitabine might be worth further study. Long-term follow-up is feasible in rare tumors.
• Challenges were lack of local resources and funding for experimental licenced therapies.

## 1 Introduction

Epithelial ovarian cancer (EOC) consists of several subtypes, with significant differences in their clinical behavior and molecular characteristics [1]. Mucinous epithelial ovarian cancer (mEOC) accounts for ~3–5% of ovarian cancers [[2], [3], [4]]. The proportion of EOCs considered to be mucinous varies significantly between countries (3–5% in Italy and Japan, up to 30–39% in Singapore and South Korea); partly due to difficulties in pathological diagnosis [5]. Survival rates for mEOC also differ by country [6].

Most primary mEOCs are diagnosed early with good prognoses following surgery [7,8]. However, advanced stage or recurrent mEOCs respond poorly to standard ovarian cancer chemotherapy. The relative rarity of mEOC means they are included in treatment trials with common types of EOC, potentially masking significant differences from other subtypes. Within randomized trials of ovarian cancer, advanced stage mEOC (stage III/IV disease or recurrence) treated with taxane/platinum therapy has worse progression-free survival (PFS) and overall survival (OS) than serous or other histologies [[9], [10], [11]]. Similar observations have been found in two case-control studies of patients treated with first-line platinum-based chemotherapy [12,13]; and in other retrospective studies based on stage III/IV disease [14,15] or in patients with recurrent stage I to IV disease [16,17].

Only one randomized trial (ICON3) has reported treatment comparisons specifically for mEOC patients, an exploratory subgroup analysis. 7% of 2074 patients had mEOC, with no difference in OS/PFS for paclitaxel-carboplatin versus either carboplatin or cisplatin-cyclophosphamide-doxorubicin [18]. The lack of evidence led us to establish the first randomized trial designed specifically for this subtype. This article also outlines direct experience of one of the first rare tumor trials conducted between the UK and US.

## 2 Methods

### 2.1 Study design

We conducted a multi-center phase III factorial trial, with accrual between March 2010 and August 2013 from 19 hospitals in the UK (called ‘mEOC’) and 12 hospitals in the US (GOG-0241). The two main trial objectives were to show (i) that oxaliplatin/capecitabine are more effective than standard paclitaxel/carboplatin, and (ii) that outcomes could be improved by adding bevacizumab to each of these two regimens.

### 2.2 Patients

Eligible patients had a reported histological diagnosis of primary mEOC; aged ≥18 years; newly diagnosed FIGO stage II–IV, or recurrence after stage I disease; no previous chemotherapy; ECOG performance status 0–2; and with acceptable biochemistry. Patients were excluded if they had brain metastases; synchronous endometrial cancer; malignancies other than ovarian cancer within prior 5 years; and cardiovascular disease precluding the use of bevacizumab. Patients were randomly assigned by an electronic system at the Cancer Trials Centre (UK) or GOG (US). Minimisation was used, with stratification factors: disease status (presence or absence of residual disease) and stage (new/recurrent stages II–IV, or recurrent stage I), in each country.

### 2.3 Interventions

Patients were allocated 1:1:1:1 to the treatment arms, involving first-line chemotherapy (3-weekly cycles, for 6 cycles), with or without concurrent bevacizumab, and those allocated to bevacizumab could have this as single agent maintenance therapy for 12 further cycles (Fig. S1):

• Carboplatin (AUC 5/6) and paclitaxel (175 mg/m^2^), both intravenous, day 1. [Pac-Carbo]
• Oxaliplatin (130 mg/m^2^ intravenous, day 1) and capecitabine (850 mg/m^2^ orally twice daily, days 1–14) [Oxal-Cape]
• Carboplatin, paclitaxel, and bevacizumab (15 mg/kg intravenous every 3 weeks), then bevacizumab maintenance (15 mg/kg on day 1, every 3 weeks). [Pac-Carbo-Bev]
• Oxaliplatin, capecitabine, and bevacizumab. [Oxal-Cape-Bev]

Carboplatin/paclitaxel was standard therapy for EOC at the time. We investigated Oxal-Cape regimen because of high response rates seen in colorectal cancer studies and that mEOCs exhibit ‘intestinal’ differentiation [[19], [20], [21]]. Oxaliplatin has activity in advanced ovarian cancer [13,22], and capecitabine was preferred over 5-flurouracil because it is taken orally and was increasingly used. Bevacizumab was another experimental therapy, to include an anti-VEGF therapy given promising findings in ovarian cancer trials [23,24], and colorectal cancer.

### 2.4 Assessments

Clinical examination and biochemistry were performed at baseline, 6-weekly in the first year, 3-monthly in the second year, and 6-monthly during years 3–5. Abdominal and pelvic CT/MRI scans were performed at baseline, the end of cycles 3 and 6, at weeks 30 and 42 post-randomization, 6-monthly in year 2, then annually in years 3–5. Health-related quality of life (QoL) was assessed using the FACT-O TOI and FACT/GOG NTX subscale. Imaging was also requested if patients had any clinical symptoms of progression, or rising biomarkers (e.g. CA125), according to local practice.

### 2.5 Histopathology

Tumor slides and the local pathology reports were reviewed centrally by specialist gynecological pathologists (RZ, WGM, RG, NW), primarily to distinguish primary ovarian from metastatic mucinous carcinomas, using various established gross and microscopic pathologic features [25,26].

Features favoring metastatic mucinous carcinoma include bilaterality, extraovarian involvement, small tumor size, involvement of the capsular surface, a multinodular pattern with intervening areas of normal ovarian parenchyma, infiltrative/destructive stromal invasion, vascular invasion particularly at the ovarian hilum and signet ring cells. While none of these features are pathognomonic for metastatic mucinous carcinoma, their presence, especially in combination, are strongly suggestive of this. Conversely, unilateral tumor, large tumor size and an expansile pattern of invasion are suggestive of a primary ovarian mucinous carcinoma. The presence of obvious benign and borderline areas is also suggestive of an ovarian primary, although ovarian metastatic mucinous carcinomas may exhibit a ‘maturation’ phenomenon with mimicry of benign and borderline neoplasia. All the trial cases exhibited extraovarian involvement at diagnosis or recurrence.

Immunohistochemistry may also assist in diagnosis, using markers such as cytokeratins 7 and 20, CEA, CA19.9, CDX2, CA125, ER, p16, SATB2 and PAX8, but these are often of limited value since most primary ovarian mucinous carcinomas exhibit ‘intestinal’ differentiation with expression of enteric markers.

### 2.6 Statistical considerations

The primary endpoint was OS, with two main comparisons: ‘oxaliplatin/capecitabine’ versus ‘no oxaliplatin/capecitabine’, and ‘bevacizumab’ versus ‘no bevacizumab’. For each main comparison, the target HR was 0.71. Assuming 5 years accrual then 18 months follow-up, we required 330 patients (259 deaths) for each comparison (80% power, two-sided 5% alpha, and 10% drop-outs).

OS was measured from randomization until death from any cause (surviving patients were censored at the date last known to be alive). PFS was measured from randomization until first evidence of disease progression or death whichever occurred first; those without an event were censored at the date they were last known to be alive. Tumor response was assessed using RECIST. CA125 response was defined as a 50% reduction from the pre-treatment CA125 value if maintained for at least 28 days [27]. The same rule was applied to CEA and CA19-9 in the absence recommended definitions of response for these markers. Asymptomatic rising levels of CA125, CA19-9 or CEA alone required radiological confirmation of progression. Toxicity was graded by the National Cancer Institute Common Terminology Criteria for Adverse Events (v4.0).

Analyses were by intention-to-treat using Cox regression for the main comparisons, and multivariable regression to explore interactions between the therapies. The worst grade of adverse event for each patient and each toxicity was used. QoL was analysed by a repeated measures mixed model.

The Independent Data Monitoring Committee stopped the trial early in 2013 because of poor accrual: only 50 patients out of the 330 target. We collected long-term data on progression, deaths and clinic visit dates (up to 21 February 2018) to observe more events.

## 3 Results

Baseline characteristics were balanced (34 UK and 16 US), Table 1. Most patients (40 of 50) completed 6 cycles of combination chemotherapy (Table S1). Among patients allocated to bevacizumab, 10 of 23 completed 12 cycles of maintenance therapy. Table S2 lists individual patients known to have received post-trial treatment (the number was similar between the four groups).

**Table 1:** Baseline characteristics.

	Paclitaxel + carboplatin *n* = 13	Oxaliplatin + capecitabine n = 13	Paclitaxel + carboplatin + bevacizumab *n* = 11	Oxaliplatin + capecitabine + bevacizumab *n* = 13
Median age years (range)	55 (32–77)	56 (20–82)	47 (29–76)	51 (28–60)
Pre-treatment CA125, median (range), IU/ml	46 (8–177)	31 (9–218)	23 (17–370)	21 (8–336)
Performance status				
ECOG 0	8	10	5	11
ECOG 1	5	3	5	2
ECOG 2	0	0	1	0
FIGO stage				
II	4	3	4	4
III	6	6	5	8
IV	1	1	1	1
Recurrent stage I^a^	2	3	1	0
Confirmed mEOC^b^	7 (43%)	1 (8%)	3 (27%)	7 (54%)

a No adjuvant chemotherapy given.

b After central pathology review.

### 3.1 Tumor response

The complete/partial RECIST tumor response rate, among those who were evaluable, was 27% (3/11) using Oxal-Cape, 40% (2/5) Oxal-Cape-Bev, 43% (3/7) Pac-Carbo-Bev, and 22% (2/9) Pac-Carbo. There were two complete responders with Oxal-Cape, and one in each of the other three groups. The number of patients with stable disease was: *n* = 5 Oxal-Cape, *n* = 3 Oxal-Cape-Bev, *n* = 0 Pac-Carbo-Bev, and *n* = 1 Pac-Carbo. The response rates were 31% (5/16) versus 31% (5/16) for any Oxal-Cape versus any Pac-Carbo (*p* = 0.65); and 42% (5/12) versus 25% (5/20) for bevacizumab versus no bevacizumab (*p*-value = 0.27).

A similar number of patients had a CA125-response in each arm (where assessable): 45% (5/11) Oxal-Cape, 50% (6/12) Oxal-Cape-Bev, 54% (6/11) Pac-Carbo-Bev, and 58% (7/12) Pac-Carbo. Among these 24 responders, the time from randomization until CA125-response was 63, 43, 43 and 34 days respectively. For the main comparisons, the CA125-response rates were 48% (11/23) versus 56% (13/23) for any Oxal-Cape versus any Pac-Carbo (*p*-value = 0.38); and 52% (12/23) versus 52% (12/23) for bevacizumab versus no bevacizumab (p-value = 0.62). There was no difference in CA125 levels for either main comparison when using patients' repeated measures over time (all *p*-values ≥ 0.42).

CEA and CA19-9 levels were similar over time for patients. Using the same definition of response as with CA-125 (acknowledging the limitations of this), the number who had a CEA-response was: 1/8 Oxal-Cape, 1/9 Oxal-Cape-Bev, 2/10 Pac-Carbo-Bev, and 0/8 Pac-Carbo. The corresponding numbers for CA19-9 response were: 1/9 Oxal-Cape, 4/9 Oxal-Cape-Bev, 2/9 Pac-Carbo-Bev, and 3/9 Pac-Carbo.

### 3.2 Progression-free and overall survival

After a median follow-up of 59 months, 34 patients had progressed or died, and 31 had died (mainly disease progression).

**Fig. 1:**
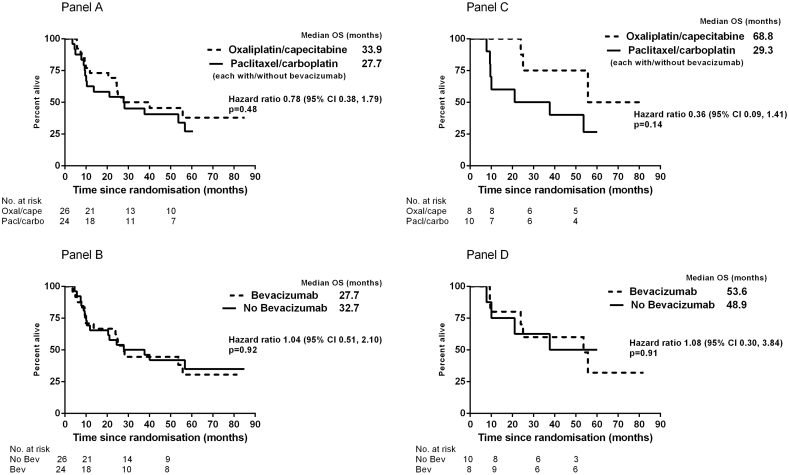
Overall survival according to the two main protocol-defined comparisons, ‘oxaliplatin + capecitabine versus no oxaliplatin + capecitabine’ and ‘bevacizumab versus no bevacizumab’, for all 50 patients (Panels A and B), and for patients with confirmed mEOC after central pathology review (Panels C and D). There was no evidence of an interaction between these two main experimental regimens (Panels A & B); interaction *p* = 0.37 for OS.

Neither of the two experimental regimens, i.e. Oxal-Cape or bevacizumab, clearly improved OS (Fig. 1) or PFS (Fig. S2); all *p*-values ≥ 0.70. OS was numerically superior for Oxal-Cape compared to Pac-Carbo (median 33.9 versus 27.7 months, HR 0.77, *p* = 0.48). Similarly, for PFS using bevacizumab (median 18.1 vs. 8.8 months; HR 0.87, *p* = 0.70).

Table 2 shows summary PFS and OS results. For all 50 patients, the median PFS was 16.4 months, somewhat higher than in previous randomized trials (7.6–11.4 months) [[9], [10], [11]]. The overall median OS (27.8 months) was also higher than in those trials (14.6–21.6 months) [[9], [10], [11]]. The 36-month rates for OS/PFS were also higher than expected (~45–55%), though with wide 95%CIs. Potential differences in patients, as well as improvements in management over time, should be acknowledged when comparing our outcomes with previous findings.

**Table 2:** Efficacy summary.

	Progression-free survival			Overall survival		
	Median months	At 12 months % (95%CI)	At 36 months % (95%CI)	Median months	At 12 months % (95%CI)	At 36 months % (95%CI)
All patients randomized (*n* = 50)						
All 4 groups together	16.4	52 (38–66)	42 (28–56)	27.8	68 (55–81)	47 (33–61)
Paclitaxel + carboplatin^a^	36.1	54 (27–81)	54 (27–81)	37.6	61 (34–87)	54 (27–81)
Oxaliplatin + capecitabine^a^	7.4	31 (6–56)	31 (6–56)	27.8	69 (44–94)	46 (19–73)
Paclitaxel + carboplatin + bevacizumab^a^	15.4	54 (24–83)	36 (8–64)	27.7	64 (35–92)	33 (4–62)
Oxaliplatin + capecitabine + bevacizumab^a^	23.2	69 (44–94)	46 (19–73)	55.7	77 (54–99)	54 (27–81)
Primary comparisons						
Oxaliplatin + capecitabine (±bevacizumab)	14.2	50 (31–69)	38 (19–57)	33.9	73 (56–90)	50 (31–69)
Paclitaxel + carboplatin (±bevacizumab)	16.4	54 (34–74)	46 (26–66)	27.7	63 (44–82)	45 (25–65)
Bevacizumab^b^	18.1	62 (43–81)	41 (21–61)	27.7	71 (53–89)	44 (24–64)
No bevacizumab^c^	8.8	42 (23–61)	42 (23–61)	32.7	65 (47–83)	50 (31–69)

Confirmed mEOC (*n* = 18)						
All 4 groups together	29.6	61 (38–83)	50 (27–73)	53.6	78 (59–97)	61 (38–83)
Primary comparisons						
Oxaliplatin + capecitabine (±bevacizumab)	38.6	75 (45–100)	50 (15–85)	68.8	100	75 (45–100)
Paclitaxel + carboplatin (±bevacizumab)	23.1	50 (19–81)	50 (19–81)	29.3	60 (30–90)	50 (19–81)
Bevacizumab^b^	33.7	70 (41–98)	50 (19–81)	53.6	80 (55–100)	60 (30–90)
No bevacizumab^c^	23.1	50 (15–85)	50 (15–85)	48.9	75 (45–100)	62 (28–95)

CI = Confidence Intervals.

a Logrank test between all 4 arms: *p* = 0.72 PFS, p = 0.70 OS.

b Paclitaxel + carboplatin + bevacizumab and oxaliplatin + capecitabine + bevacizumab.

c Paclitaxel + carboplatin and oxaliplatin + capecitabine.

We were particularly interested in the effect of adding bevacizumab to Pac-Carbo (pre-specified analyses). The HRs were 1.12 (*p* = 0.82) and 1.47 (*p* = 0.44) for PFS and OS respectively (Pac-Carbo-Bev versus Pac-Carbo), i.e. no benefit. Similarly, for adding bevacizumab to Oxal-Cape, the HRs were 0.55 (*p* = 0.23) and 0.77 (*p* = 0.61) for PFS and OS respectively (Oxal-Cape-Bev versus Oxal-Cape).

Fifteen of the 50 patients (30%) had at least 48 months without progression; 7 had stage III disease at diagnosis (Table 3). Notably, 9 patients were given Oxal-Cape (35%: 9/26) compared to 25% (6/24) given Pac-Carbo (each with/without bevacizumab). Some of these 15 patients might have lacked macroscopic residual disease after surgery.

**Table 3:** Individual patients without progression by 48 months from randomization (total number randomized patients in each group is shown).

Patient number	Progression-free time, months	Age	FIGO stage	Performance status	No. of chemo cycles	No. of maint cycles	Confirmed mEOC^b^
Paclitaxel + carboplatin (n = 13)							
1^c^	50	53	II	0	3		No
2	55	58	III	0	6		Yes
3	59	60	II	1	6		Yes
4	60	55	III	0	6		No

Oxaliplatin + capecitabine (n = 13)							
5	51	57	III	0	6		No
6	52	61	II	0	6		Not known
7	63	54	Rec^a^	0	6		Not known
8	85	26	III	0	6		No

Paclitaxel + carboplatin + bevacizumab (n = 11)							
9	50	47	II	0	6	0	No
10^e^	60	55	II	1	6	12	No

Oxaliplatin + capecitabine + bevacizumab (n = 13)							
11^d^	54	31	III	0	6	12	Yes
12	54	50	III	0	6	12	Yes
13	61	48	II	0	6	1	Yes
14	71	58	III	1	6	4	No
15	82	58	II	1	6	12	Yes

Chemo = chemotherapy.

Maint = maintenance with bevacizumab.

a Recurrence after stage I; no adjuvant chemotherapy given.

b After central pathology review.

c Died at 57 months.

d Died at 56 months.

e Had doxorubicin/cyclophosphamide 26 months after ending trial treatment (all other patients in the table had no record of subsequent treatment after completing the trial treatment).

### 3.3 Adverse events and QoL

The percentage of patients who experienced any grade 3–4 toxicity was: 61% (8/13) Oxal-Cape, 85% (11/13) Oxal-Cape-Bev, 54% (6/11) Pac-Carbo-Bev, and 61% (8/13) Pac-Carbo (Tables 4 and S3). As expected, hypertension was more common among patients receiving bevacizumab. Alopecia was more common in those given paclitaxel: 83% (20/24) paclitaxel versus 23% (6/26) no paclitaxel.

**Table 4:** Clinically relevant grade 3–4 adverse events. Number of patients for each type of event, maximum grade per patient. (All adverse events set out in Supplemental Table S1).

	Paclitaxel + carboplatin *n* = 13	Oxaliplatin + capecitabine n = 13	Paclitaxel + carboplatin + bevacizumab *n* = 11	Oxaliplatin + capecitabine + bevacizumab n = 13
Allergic reaction	1	1	.	.
Bleeding	.	.	.	2
Constipation	.	.	.	1
Diarrhoea	.	1	.	3
Dyspnoea	.	1	.	.
Fatigue	.	.	1	.
GI perforation	.	.	.	1
Hand-foot syndrome	.	.	.	2
Hypertension	.	4	3	6
Nausea/vomiting	.	.	.	2
GI, other	.	1	.	.
Pain	1	.	1	1
Peripheral sensory neuropathy	2	.	.	1
Pneumothorax	.	1	.	.
Rash	.	1	.	.
Thromboembolic event	.	.	1	.
Vaginal bleeding	.	.	.	1
Anaemia	1	.	.	2
Low lymphocytes	.	.	1	.
Low neutrophils	5	1	1	.
Low platelets	2	.	.	.
Low white blood cells	1	.	1	.
Hypomagnesemia	.	.	1	.
Abnormal laboratory values	.	.	1	1
Any grade 3–4 event^a^	8 (61%)	8 (61%)	6 (54%)	11 (85%)

a Each patient counted only once.

QoL was similar across all treatment groups for physical and functional well-being, and neurotoxicity (Table S4), with small differences in total scores for both main comparisons (Table S5).

### 3.4 Central pathology review

Tumor material was available for retrospective central pathology review in 40 of 50 cases. 18 (45%; 18/40) were confirmed as having primary mEOC; the others were considered to represent metastatic disease, usually from the upper/lower gastrointestinal tract or cervix.

There was no clear evidence that patients with confirmed primary mEOCs had different outcomes than those considered to have metastatic neoplasms: HRs for primary mucinous versus metastatic tumors were 1.03 (95%CI 0.48–2.19, *p* = 0.94) and 0.74 (95%CI 0.33–1.66, *p* = 0.46) for PFS and OS respectively, adjusted for the two main treatment comparisons and FIGO stage.

Table 2 summarises PFS/OS for the 18 with confirmed primary mEOCs, and Figs. 1 and S2 show Kaplan-Meier curves. The number of patients is too small to make any reliable conclusions. The PFS and OS HRs for bevacizumab versus no bevacizumab were 0.76 (*p* = 0.62) and 1.08 (*p* = 0.91) respectively. However, the PFS HR for Oxal-Cape compared to Pac-Carbo (both with or without bevacizumab) was 0.62 (*p* = 0.40), while the OS curves were clearly separated in favor of Oxal-Cape, with HR 0.36 (*p* = 0.14).

## 4 Discussion

The mEOC/GOG-0241 trial is one of the first US-UK collaborations to establish rare tumor trials, and a precursor to the International Rare Cancers Initiative [28]. We set up and conducted a randomized controlled trial in a rare subtype, although only recruited 50 patients. We also showed the value of undertaking long-term follow-up. There were two separate sponsors (US and UK) because of significant issues associated with having single or joint sponsorship for transatlantic studies, such as processing of contracts, costs, implications for Roche who provided bevacizumab, and insurance.

No firm conclusions about best treatment options can be made. However, we show slight evidence that oxaliplatin/capecitabine could be investigated further. Our trial also provides estimates of OS/PFS within a contemporary cohort of mEOC patients using high quality data, including pathologically confirmed mEOC. The 3-year OS/PFS rates seemed higher than in previous studies [10], with a notable number of long-term survivors without progression (Table 3).

Given the limited scientific impact of our trial, we focus our discussion on the reasons why this study of a rare tumor failed. Few investigators have provided detailed information about their experiences of conducting rare cancer trials. For example, in an international trial of high-grade uterine leiomyosarcoma published in November 2018 in the Journal of Clinical Oncology, only 38 patients were recruited out of the target of 216, but there was a lack of reasons for the poor accrual [29]. Therefore, our intention here in our paper is that important lessons can be learnt, for the benefit of other researchers and funders.

Two trial-specific issues were encountered. Firstly, there were difficulties with local pathology evaluation. Only 45% of patients were considered to have primary mEOCs on specialist central review, similar to 30% found by Zaino and colleagues [30]. Kommoss and colleagues showed the impact of revised histopathological diagnostic approaches, where one pathologist reviewed 23 mEOC cases in 2002, but only 9 were classified as primary mEOC when reviewed again in 2014 using WHO criteria (blinded to the original diagnosis) [31]. Diagnostic criteria are more standardized now with high inter-observer reproducibility when classifying ovarian carcinomas. The second issue was the declining incidence of primary mEOC over time (from 10–12% to 3–5% currently), predominantly attributable to revised pathological assessment and the realization that many advanced stage mEOCs represent metastases from extraovarian sites [32].

Funders strongly encourage and provide significant funding specifically for uncommon/rare cancer trials, but our experience should increase their awareness of some major practical difficulties. Firstly, individual sites often consider the resources required for setting up and conducting therapy trials, including contracts, to be disproportionately high compared to the low number of patients they expect to recruit. This is particularly problematic for non-commercial studies, who usually do not provide initial set-up payments plus per patient costs (unlike industry-sponsored trials). There were reports that those responsible for the local research budgets in the UK were unwilling to support the trial for financial/resource reasons, while prioritising trials in commoner cancers.

Secondly, several sites considered that oxaliplatin/capecitabine were not a recommended treatment for mEOC, and so not prepared to fund them locally. This was particularly disappointing because both drugs are easily available, and with a low treatment cost so it would have a tiny impact on an individual site's total anti-cancer therapy budget, especially since most sites would recruit very few (1–3) patients per year. When mEOC/GOG0241 started, the cost of oxaliplatin/capecitabine was ~£433 per cycle (£2598 for 6 cycles), or $692 per cycle ($4156 for 6 cycles). The issue of funding ‘experimental’ treatments has a potential detrimental effect on precision medicine strategies and trials using molecular targets for patient eligibility. Our experience suggests that unless the targeted agent is funded/provided by industry, local investigators could have difficulty persuading their funders to pay for necessary trial drugs. In MEOC/GOG-0241, Roche provided bevacizumab so there was no issue here, but the trial grants which came from the NCI in the US and Cancer Research UK in the UK do not fund treatments, hence we were completely reliant on hospitals to provide oxaliplatin/capecitabine.

A third issue was lack of support from investigators. In 2013, we surveyed all active oncology trial sites in the US and UK, but only 57 out of 217 responded. We also surveyed 700 attendees at an annual Gynecologic Oncology Group meeting, in which only 54 responded but about half of the respondents believed the trial was still relevant. The low survey response rates suggested an overall lack of interest to proceed with the trial locally. We believe that this was largely due to the site problems raised above, because when the idea for the trial was first initiated before 2008 there was strong international support for it, which is why it proceeded to successful funding applications in both the US and UK.

Further evidence for these issues came from examining the number of activated and recruiting centers. In the UK, 41 hospitals were activated of which 19 recruited; only 7 each recruited ≥2 patients, and the rest each had one patient only. The US situation was worse, with only 12 out of 176 activated sites enrolling patients; only two each recruited ≥2 patients, and 10 each had only one patient. If every activated center had recruited only two patients during the entire accrual period of 3.5 years, we would have 434 patients, including ~156 with confirmed primary mEOC. This would have been a major achievement and provided sufficient data to help manage mEOC patients, particularly given the factorial trial design.

Therapies are still typically based on organ of origin rather than on molecular/genetic characteristics. Gene expression arrays show that mucinous cancers are distinctly different from other subtypes [33]. About 50% of mucinous tumors have KRAS mutations and 20% HER2 amplification, higher than with serous carcinomas, and both markers generally seem associated with better survival and fewer recurrences [31,[34], [35], [36]]. We assumed that mEOC tumors would respond in the same favorable way to oxaliplatin-capecitabine as colorectal carcinomas, since both exhibit intestinal differentiation [[19], [20], [21]]. However, while they share some common molecular alterations, they differ in others: HER2 amplification and KRAS mutations are more common in mEOC while BRAF, APC and CTNNB1 mutations are less common [37]. Potential treatment approaches for mEOC might include immunotherapy [38], radiotherapy [39], combination PI3K/mTOR and MEK inihibitors [40], and oxaliplatin-dasatinib [41].

mEOC patients are disadvantaged because treatment regimens for them do not have the same level of evidence as for other cancers. The Fifth Ovarian Cancer Consensus Conference [42] concluded that recruiting mEOC patients to trials remains important. In future studies, central pathology review should be part of the eligibility criteria, possibly facilitated by a review panel utilising digital technology, as successfully adopted in the GOG281 trial. However, while other ovarian subtypes can be readily diagnosed using a single histological section, the diagnosis of primary mEOC is generally more problematic, requiring multiple sections to distinguish primary ovarian from metastatic neoplasms.

A key lesson learnt from mEOC/GOG-0241 is that international collaboration and well-intentioned support from research funders may be insufficient for a successful trial in rare cancers. Two trials under the International Rare Cancers Initiative have closed (NCT02051868 and NCT01979523): one completed, and the other did not open in Europe after all and stopped early. It would be important for these investigators to report difficulties encountered. During mEOC/GOG-0241, problems at sites included funding, prioritisation and administrative burden. Future investigators attempting to establish rare tumor trials should address these issues early and directly.

Conducting traditional randomized phase III studies are challenging for rare tumors, and this is even more so with biomarker-directed therapies within precision medicine research. We therefore need better ways of evaluating experimental therapies for rare tumors such as mEOC and also producing useful data to help guide treatments. This may simply involve well conducted single-arm or small randomized phase II trials, or adaptive designs [43]. An alternative is to take a ‘big data’ approach and prospectively collect data in a systematic manner, as well as using the information that already resides within the databases of every major cancer centre, and couple it with central pathology review and molecular analysis. Using real-world data could act as controls for single arm trials of experimental therapies. Finally, we believe that better progress can be made in treating rare tumors if there is a closer connect between research funders and local decision-makers.

## Appendix A Supplementary data

Supplementary material Image 1
