# Supplementary material for: An international, phase III randomized trial in patients with mucinous epithelial ovarian cancer (mEOC/GOG 0241) with long-term follow-up: and experience of conducting a clinical trial in a rare gynecological tumor
Source: Gynecol Oncol. 2019 Jun;153(3):541–8. doi: 10.1016/j.ygyno.2019.03.256 (PMC6559214; doi:10.1016/j.ygyno.2019.03.256)
Supplement: Supplementary file 1 — Supplementary material [file mmc1.docx]

**Supplementary Table S1. Number of trial treatment cycles received by patients**

| No. cycles completed | Paclitaxel +carboplatin  n=13 | Oxaliplatin +capecitabine  n=13 | Paclitaxel +carboplatin +bevacizumab  n=11 | Oxaliplatin +capecitabine +bevacizumab  n=13 |
| --- | --- | --- | --- | --- |
| **Chemotherapy** |  |  |  |  |
| 2 | 2 |  |  | 1 |
| 3 | 2 |  | 1 | 1 |
| 4 | 1 | 1 |  |  |
| 5 |  | 1 |  |  |
| 6 | 8 | 11 | 10 | 11 |
|  |  |  |  |  |
| **Maintenance bevacizumab** | |  |  |  |
| None received |  |  | 3 | 2 |
| 1-2 |  |  |  | 3 |
| 4 |  |  | 3 | 1 |
| 5 |  |  | 1 |  |
| 6 |  |  |  | 1 |
| 12 |  |  | 4 | 6 |

Protocol-specified 6 cycles of combination chemotherapy, then up to 12 cycles of bevacizumab for those allocated to maintenance therapy

**Supplementary Table S2. Subsequent therapy after completion of trial treatment in each of the four randomized groups and outcomes. Data collected up to 25 months after completion of trial treatment.**

| Patient number and treatment | | Months post trial treatment * | Progression, no. of months post randomisation | Death no. of months post randomisation |
| --- | --- | --- | --- | --- |
| **Trial arm: Paclitaxel+carboplatin** | |  |  |  |
| 1 | Oxaliplatin+fluorouracil | 2.7 | 4.6 | 7.7 |
| 2 | Gemcitabine | 7.1 | 10.1 | 21.0 |
| 3 | Oxaliplatin+capecitabine | 1.1 | 2.8 | 8.8 |
| 4 | Oxaliplatin+capecitabine+bevacizumab | 0.9 | 2.0 | 10.0 |
|  |  |  |  |  |
| **Trial arm: Oxaliplatin+capecitabine** | |  |  |  |
| 5 | Carboplatin+paclitaxel  Liposomal doxorubicin+carboplatin | 0.1-18.0 | 4.5 | 24.5 |
| 6 | Bevacizumab+capecitabine+irinotecan  Oxaliplatin+capecitabine | 6.1-18.5 | 4.4 | 40.0 |
| 7 | Carboplatin+paclitaxel  Bevacizumab+gemcitabine+ carboplatin | 5.5-18.4 | 3.8 | 39.9 |
| 8 | Dasatinib  Cisplatin+prexasertib  Demicizumab+paclitaxel  Pegylated lipsosomal doxorubicin  Niraparib | 6.7-18.6 | 10.3 | 27.8 |
| 9 | Cabozantinib | 2.3 | 5.6 | 9.3 |
|  |  |  |  |  |
| **Trial arm: Oxaliplatin+capecitabine+bevacizumab** | |  |  |  |
| 10 | Carboplatin+paclitaxel | 0.8 | 1.4 | 5.7 |
| 11 | Carboplatin+paclitaxel | 4.4 | 9.1 | 25.0 |
| 12 | Carboplatin+paclitaxel  Bevacizumab+capecitabine | 7.0-8.2 | 18.1 | 24.8 |
| 13 | Liposomal doxorubicin | 16.5 | 23.2 | 23.9 |
|  |  |  |  |  |
| **Trial arm: Paclitaxel+carboplatin+bevacizumab** | |  |  |  |
| 14 | Carboplatin+paclitaxel | 5.8 | 17.4 | 28.2 |
| 15 | Oxaliplatin+capecitabine  Oxaliplatin+fluorouracil  Cisplatin | 1.1-5.7 | 6.8 | 13.6 |
| 16 | Doxorubicin+cyclophosphamide | 25.6 | Not progressed | Not died |
|  |  |  |  |  |

*no. of months after last protocol-specified trial treatment, ie chemotherapy only or chemotherapy followed by maintenance bevacizumab

**Supplementary Table S3. All adverse events. Number of patients for each type of event, maximum grade per patient. (Gd=grade)**

|  | Oxaliplatin +capecitabine  n=13 | | Oxaliplatin +capecitabine +bevacizumab  n=13 | | Paclitaxel +carboplatin  n=11 | | Paclitaxel +carboplatin +bevacizumab  n=13 | |
| --- | --- | --- | --- | --- | --- | --- | --- | --- |
|  | Gd 1-2 | Gd 3-4 | Gd 1-2 | Gd 3-4 | Gd 1-2 | Gd 3-4 | Gd 1-2 | Gd 3-4 |
| Abnormal laboratory values | 10 | . | 9 | 1 | 8 | . | 9 | 1 |
| Allergic reaction |  |  |  |  |  |  |  |  |
| Allergic rhinitis | 1 | . | 1 | . | . | . | 1 | . |
| Alopecia | 1 | . | 5 | . | 11 | . | 9 | . |
| Anaemia | 7 | . | 4 | 2 | 11 | 1 | 4 | . |
| Bleeding | 1 | . | 4 | 2 | 1 | . | 3 | . |
| Cold-like symptoms | 3 | . | 6 | . | 2 | . | 7 | . |
| Constipation | 4 | . | 9 | 1 | 3 | . | 7 | . |
| Diarrhoea | 5 | 1 | 5 | 3 | 5 | . | 5 | . |
| Dyspnoea | . | 1 | . | . | 2 | . | 3 | . |
| Edema limbs | 1 | . | . | . | . | . | . | . |
| Fatigue | 10 | . | 11 | . | 10 | . | 7 | 1 |
| Fever | . | . | 1 | . | 3 | . | 2 | . |
| GI perforation | . | . | . | 1 | . | . | . | . |
| Hand-foot syndrome | . | . | 5 | 2 | 1 | . | . | . |
| Headache | 4 | . | 4 | . | 3 | . | 5 | . |
| Hypertension | 4 | 4 | 4 | 6 | 3 | . | 6 | 3 |
| Hypomagnesemia | . | . | . | . | 1 | . | 2 | 1 |
| Infection | . | . | 3 | . | 4 | . | 3 | . |
| Laryngeal spasm | 4 | . | . | . | . | . | . | . |
| Low lymphocytes | 1 | . | . | . | 1 | . | 1 | 1 |
| Low mood | 2 | . | 1 | . | 1 | . | 1 | . |
| Low neutrophils | 4 | 1 | 7 | . | 2 | 5 | 3 | 1 |
| Low platelets | 3 | . | 7 | . | 6 | 2 | 5 | . |
| Low white blood cells | 5 | . | 6 | . | 4 | 1 | 3 | 1 |
| Mucositis | 1 | . | 3 | . | 1 | . | 1 | . |
| Nausea/vomiting | 8 | . | 8 | 2 | 8 | . | 9 | . |
| Oral problems | 2 | . | 9 | . | 3 | . | 4 | . |
| GI, other | 5 | 1 | 6 | . | 8 | . | 7 | . |
| Pain | 6 | . | 7 | 1 | 8 | 1 | 6 | 1 |
| Peripheral sensory neuropathy | 11 | . | 9 | 1 | 9 | 2 | 8 | . |
| Pneumothorax | . | 1 | . | . | . | . | . | . |
| Raised CA19-9 | . | . | . | . | . | . | 2 | . |
| Raised blood counts | 1 | . | 1 | . | 1 | . | . | . |
| Rash | 1 | 1 | 4 | . | 4 | . | 3 | . |
| Stomatitis | 2 | . | . | . | . | . | 1 | . |
| Taste Alteration | 2 | . | 2 | . | 3 | . | 1 | . |
| Thromboembolic event | . | . | . | . | . | . | . | 1 |
| Urinary problems | . | . | 2 | . | 1 | . | 1 | . |
| Vaginal bleeding | 1 | . | 1 | 1 | . | . | 1 | . |
| Weight gain | 1 | . | 2 | . | . | . | 1 | . |
| Weight loss | 3 | . | 5 | . | 2 | . | 4 | . |
| Other | 4 | . | 7 | . | 4 | . | 7 | . |

**Supplementary Table S4. Health-related quality of life using the last recorded measurement for each patient, excluding QoL measures for patients whilst on bevacizumab maintenance. Data expressed as the mean (standard error) for each group. Total score is the sum of all four domains, provided ≥80% of the survey items were completed by the patient. High score indicates good health.**

|  | Paclitaxel +carboplatin  n=10 | Oxaliplatin +capecitabine  n=13 | Paclitaxel +carboplatin +bevacizumab  n=9 | Oxaliplatin +capecitabine +bevacizumab  n=9 |
| --- | --- | --- | --- | --- |
|  |  |  |  |  |
| Physical well-being | 23.1 (1.5) | 23.6 (0.9) | 19.8 (2.3) | 20.9 (1.7) |
| (range 0-28) |  |  |  |  |
|  |  |  |  |  |
| Functional well-being | 19.9 (1.8) | 19.3 (2.0) | 18.0 (2.5) | 17.9 (2.3) |
| (range 0-28) |  |  |  |  |
|  |  |  |  |  |
| Additional concerns* |  |  |  |  |
| (range 0-44) | 33.6 (1.7) | 34.5 (1.5) | 31.2 (2.7) | 35.4 (1.9) |
|  |  |  |  |  |
| Neurotoxicity |  |  |  |  |
| (range 0-16) | 10.5 (1.7) | 12.3 (0.9) | 11.8 (1.9) | 10.1 (2.0) |
|  |  |  |  |  |
| Total score | 87.1 (5.5) | 89.4 (4.5) | 80.7 (8.8) | 84.3 (5.9) |
| (range 0-116) |  |  |  |  |
|  |  |  |  |  |

### *includes gastrointestinal toxicity, hair loss, appearance, interest in sex

**Supplementary Table S5. Mean difference in QoL scores for** **the two main protocol-defined comparisons*, from a mixed modelling/repeated measures analysis, based on all the QoL measurements for each patient, excluding QoL measures for patients whilst on bevacizumab maintenance.**

|  | Mean difference (95% CI) | p-value |
| --- | --- | --- |
|  |  |  |
| **Oxaliplatin/capecitabine (± maintenance) vs paclitaxel/carboplatin (± maintenance)** | | |
| Physical well-being  (range 0-28) | 1.5 (-0.6, 3.7) | 0.16 |
| Functional well-being  (range 0-28) | 0.04 (-2.8, 2.8) | 0.98 |
| Additional concerns*  (range 0-44) | 1.7 (-1.1, 4.5) | 0.24 |
| Neurotoxicity  (range 0-16) | -0.8 (-2.4, 0.8) | 0.33 |
| Total score  (range 0-116) | 2.6 (-4.9, 10.2) | 0.49 |
|  |  |  |
| **Bevacizumab maintenance versus no maintenance** | |  |
| Physical well-being | -1.2 (-3.4, 1.1) | 0.30 |
| Functional well-being | -0.5 (-3.4, 2.3) | 0.72 |
| Additional concerns* | -0.6 (-3.5, 2.3) | 0.68 |
| Neurotoxicity | 0.1 (-1.5, 1.8) | 0.90 |
| Total score | -2.6 (-10.3, 5.1) | 0.50 |
|  |  |  |

*‘oxaliplatin+capecitabine versus no oxaliplatin+capecitabine’ and ‘bevacizumab versus no bevacizumab’

*includes gastrointestinal issues, hair loss, appearance, and interest in sex

Total score is the sum of all the other four domains (as long as ≥80% of the survey items were completed by the patient)

A positive mean difference indicates better QoL health in favor of oxaliplatin/capecitabine (top part of the table), or in favor of bevacizumab maintenance (bottom part of the table).

**Supplementary Figure S1. Flow chart of the mEOC trial/GOG024**

**Supplementary Figure S2. Progression-free survival according to the two main protocol-defined comparisons, ‘oxaliplatin+capecitabine versus no oxaliplatin+capecitabine’ and ‘bevacizumab versus no bevacizumab’, for all 50 patients (Panels A and B) and for patients with confirmed mEOC after central pathology review (Panels C and D)****. There was no evidence of an interaction between these two main experimental regimens (Panels A & B); interaction p=0.37 for PFS.**
